# Epidemiology of *Mycoplasma genitalium* and *Trichomonas vaginalis* in the primary health care setting in the Netherlands

**DOI:** 10.1017/S095026882300064X

**Published:** 2023-05-05

**Authors:** Erlangga Yusuf, Kelly Mertens, Nico van Lisdonk, Cindy Houwen, Khoa T. D. Thai

**Affiliations:** 1 Star-shl Medical Diagnostic Center, Rotterdam, The Netherlands; 2 Department of Medical Microbiology and Infectious Diseases, Erasmus MC, Rotterdam, The Netherlands

**Keywords:** *Mycoplasma genitalium*, PCR, prevalence, primary health care, sexually transmitted, diseases, *Trichomonas vaginalis*

## Abstract

The aim of this paper is to describe the prevalence of *Mycoplasma genitalium* and *Trichomonas vaginalis* in patients who visited general practitioners in the Netherlands. Additionally, we describe the prevalence of *M. genitalium* resistance to azithromycin and moxifloxacin. We used data from 7,411 consecutive female patients who were screened for *Chlamydia trachomatis*, *Neisseria gonorrhoeae*, *M. genitalium*, and *T. vaginalis* and data from 5,732 consecutive male patients screened for *C. trachomatis*, *N. gonorrhoeae*, and *M. genitalium*. The prevalence of *M. genitalium* and *T. vaginalis* in female patients was 6.7% (95% CI: 6.2 to 7.4) and 1.9% (95%CI: 1.6 to 2.2%), respectively. *M. genitalium* prevalence in male patients was 3.7% (3.3 to 4.3). *M. genitalium* co-occurred with *C. trachomatis* in 1.4% (0.3 to 0.6%) of female and in 0.7% (0.5 to 0.9) of male patients. Macrolide resistance gene mutations and fluoroquinolone resistance gene mutations were detected in 73.8% and 9.9%, respectively. We concluded that *M.genitalium* is relatively infrequently found in a large general practitioner population in the Netherlands. It can co-occur with *C. trachomatis*, and is often resistant to azithromycin. Therefore, when treating sexually transmitted infections, these prevalence and resistance data should be taken into account.

## Introduction

Sexually transmitted infections (STIs) have a significant impact on quality of life and reproductive health. The rates of STIs have increased substantially worldwide, including in high-income countries during the 21st century [1]. There are more than 30 different bacteria, viruses, and parasites that can be transmitted through sexual contact [2]. While surveillance data are widely available for *Chlamydia trachomatis* and *Neisseria gonorrhoeae*, the epidemiology of *Mycoplasma genitalium* and *Trichomonas vaginalis* is less known [3, 4]. A meta-analysis published in 2018 included only two studies from Europe on the prevalence of *M. genitalium* in the general population, and three studies on men who have sex with men (MSM) who visited a community-based clinic in Europe. [5]. Arguably, the prevalence of STI pathogens can be affected by a variety of reasons such as the study setting, the structure of the health care system, and whether the individuals with STI complaints are advised to go to primary care or to an STI clinic. In the general population of British individuals who did not exhibit symptoms of STI, the prevalence of *M. genitalium* was 1.2% in men and 1.3% in women [6]. In patients who underwent STI screening in France, the prevalence was comparable, that is 1.7% [7]. In the same French study, the prevalence of *T. vaginalis* was 3.4%. In the USA, the *T. vaginalis* positivity rate in a population-based study was reported at 2.3% among adolescents [8] and 3.1% among reproductive-age women [9]. There is also paucity of data on the co-occurrence of *M. genitalium* and *T. vaginalis* with *C. trachomatis* and *N. gonorrhoeae.*

In the Netherlands, there is limited data on the prevalence of *M. genitalium* and. *T. vaginalis*, and the available data come from a mixed population. In a study of samples taken from primary care and hospital care settings, the prevalence of *M. genitalium* was 1.9% and *T. vaginalis* was 0.6% [10]. Another study conducted in the Netherlands that collected samples from STI clinics, general practitioners (GPs), and hospitals found *M. genitalium* and *T. vaginalis* prevalence of 1.4% and 4.5%, respectively [11]. Additional data are required on the epidemiology of *M. genitalium* and *T. vaginalis* in the primary care population of the Netherlands.

Therefore, the aim of this study is to describe the epidemiology of *M. genitalium* and *T. vaginalis*, especially in light of their co-occurrence with STI pathogens *C. trachomatis* and *N. gonorrhoeae* in patients who visited GPs in the Netherlands. As a second objective, we sought to describe the prevalence of *M. genitalium* genotypically resistant to azithromycin and fluoroquinolones.

## Materials and methods

### Study setting and population

The data used in this study were derived from specimens (pharyngeal-, urethral- (only in men), and vaginal swabs (only in women) and urine) submitted to the Star-shl Medical Laboratory during a four-year period (between January 2018 and December 2021). The specimens were obtained from patients who visited GPs due to urethral symptoms (that is dysuria or discharge) or vaginal symptoms (that is dysuria, discharge, or vaginal blood loss), or from their sexual contacts. The Star-shl Medical Laboratory provides services for 1,500 GPs, mostly from the southwestern region of the Netherlands. This region has an estimated population of 3.5 million inhabitants. In the whole country, there are around 12,000 GPs [12], and people can go to either their GP or the Municipal Public Health Service (GGD in Dutch) for an STI test. STI testing offered by the GGD is anonymous and frequently sought by high-risk patients, such as men who have sex with men (MSM) and sex workers. The present study relied solely on laboratory data, so approval from the ethical committee was not required.

### Sampling and molecular testing

Swabs and first-void urine were collected and transferred to appropriate sample collection media, and transported to the laboratory within the same day. For the detection of *M. genitalium* and *T. vaginalis*, Aptima™ *M. genitalium* [13] and Aptima™ *Trichomonas vaginalis* assay [14] (both Hologic Inc, San Diego, USA), respectively, were used. The Aptima™ Combo 2 (Hologic Inc, San Diego, USA) was used for the detection of *C. trachomatis* and *N. gonorrhoeae* [15]. These assays were performed in Panther systems (Hologic Inc, San Diego, USA), and are in-vitro diagnostics that involve the capture of rRNA target molecules and transcription-mediated amplification of specific regions.

Among samples from patients who tested positive for *M. genitalium*, 202 samples were randomly selected and tested for macrolide resistance using the Allplex™ MG & AziR assay that allowed detection of any of six point mutations in region V of the 23S rRNA gene (A2058C, A2058G, A2058T, A2509C, A2509G, A2509T) that may be responsible for azithromycin resistance [16]. In the same samples, fluoroquinolone resistance was tested using the Allplex MG™ & MoxiR assay that detects single nucleotide polymorphisms (SNPs) in the *par*C gene: A247C, G248A, G248T, G259A, G259C, G259T [16]. The mutation detection assays were from Seegene (Seoul, South Korea) and performed according to the manufacturer’s instructions. If more than one sample was positive for *M. genitalium*, only one sample per patient was randomly chosen for these antibiotic resistance tests.

### Statistical analysis

Statistical analyses were conducted in Rstudio (version 2022.07.1), using R version 4.2.1. We calculated and presented the proportion of patients who tested positive for the STI pathogens investigated in this study (including the percentage and their 95% confidence interval, 95% CI). We also calculated the proportion of patients with a co-occurrence of *M. genitalium* or *T. vaginalis* with *C. trachomatis* and *N.* *gonorrhoeae.* No formal statistical testing was performed since this study did not pose any particular hypothesis.

## Results

### Prevalence

During the study period, GPs ordered tests for all four pathogens in 7,411 female patients (median age 29.4 years (IQR 23.4 to 38.5)), and for *C. trachomatis*, *N. gonorrhoeae*, and *M. genitalium* in 5,732 male patients (30.4 years (24.5 to 40.2)).

In female patients, 7.0% (95%CI: 6.4 to 7.6) were positive for *C. trachomatis* and 0.8% (0.6 to 1.0) were positive for *N. gonorrhoea.* *M. genitalium* was positive in 6.7% (6.2 to 7.4) and *T. vaginalis* was positive in 1.9% (1.6 to 2.2).

In male patients, 5.5% (95%CI: 4.9 to 6.1) were positive for *C. trachomatis* and 1.7% (1.4 to 2.1) were positive for *N. gonorrhoea.* *M. genitalium* was positive in 3.7% (3.3 to 4.3).

*M. genitalium* co-occurred with *C. trachomatis* in 1.4% (0.3 to 0.6) of female patients and in 0.7% (0.5 to 0.9) of male patients. *T. vaginalis* co-occurred with *C. trachomatis* only in 0.2% (0.1 to 0.3) of female patients. The co-occurrence of *M. genitalium* and *N. gonorrhoea* was rarely observed, that is in 0.1% (0.04 to 0.2) of female patients, and in 0.2% (0.1 to 0.4) of male patients.

### Macrolide and fluoroquinolone resistance in M. genitalium

Macrolide resistance–associated gene mutations were detected in 149 out of 202 (73.8.2%) randomly selected *M. genitalium* positive samples. The frequency of fluoroquinolone resistance–associated gene was much lower than macrolide resistance, that is 20 out of 202 (9.9%) randomly selected *M. genitalium* positive samples. Both macrolide and fluoroquinolone gene mutations were detected in five patients. The mutations associated with macrolide and fluoroquinolone resistance found in this study are presented in Table 1.

**Table 1.** Point mutations associated with azithromycin and fluoroquinolone resistance in positive *M. genitalium* samples

	n (%)
*Macrolide resistance*	149 (73.8)
A2058G	61
A2059G	50
A2058T	25
A2509C	4
A2058C and A2059T	0
Invalid	4
*Fluoroquinolone resistance*	20 (9.9)
G248T	10
G259A	8
G248A	2
G259C, G259T, A247C	0

## Discussion

Dutch guidelines do not recommend routine screening for asymptomatic patients for *M. genitalium* and *T. vaginalis* [17, 18], and perhaps this is the reason why knowledge on the co-occurrence of these pathogens with other STIs in the Netherlands is scarce. The difference in the prevalence of STI pathogens is determined largely by the population and the test that is used. The comparison should therefore be made, for example between GP populations and mixed-study populations (e.g. hospitals, patients visiting STI clinics). Plausibly, our GP patient population will have a lower prevalence of STIs including *M. genitalium* than the population of patients who visited the GGD. Due to anonymous testing, the GGD is frequently sought by high-risk patients, such as MSM and sex workers. The prevalence of *M. genitalium* in our study population (5.5% combined male and female) is three times higher than the prevalence of *M. genitalium* in a mixed GP and hospital population in a bordering region in the Netherlands (2.1%) [10]. The prevalence of *T. vaginalis* in our study was also slightly higher than in that study (1% (combined male and female) versus 0.7%). An explanation for this could be the high sensitivity of the TMA-based Aptima assay [19] used in our study. Another study from the Netherlands using a mixed-study population (STI clinics, GPs, and hospitals), where no separate data could be derived for the GP population, showed a prevalence of *M. genitalium* and *T. vaginalis* of 4.5% and 1.4%, respectively [11]. In that study, an in-house PCR was used.

Our study confirmed a very high percentage of a genotypic macrolide resistance as also found in the study from Hetem and colleagues from the Netherlands. It was shown in that study that 66% of positive *M. genitalium* samples showed macrolide resistance–associated mutations [18]. This study was published after a systematic review and meta-analysis that showed macrolide resistance proportions of 27.5% in non-Nordic European countries. The range is however wide, from 6.5% in Belgium to 74.3% in the UK [16]. In this systematic review, the proportion of *M. genitalium* that was resistant to fluoroquinolone in non-Nordic countries was 3.2%, lower than the 9.9% found in our study.

Our findings present several new recommendations for practice. Since *M. genitalium* is occasionally found, it is recommended that it should be tested when test results for *C. trachomatis* or *N. gonorrhoeae* turn out to be negative but the patient still experiences STI symptoms. Another recommendation is that macrolide resistance should be performed for patients who retain symptoms after macrolide treatment. The number of macrolide resistance gene mutations may be high, but a discrepancy between genotypic and phenotypic resistance may occur.

Our study has several limitations. It does not collect specific clinical data, but our study was not designed to link the clinical data to STI pathogens. Another limitation is that we only performed macrolide or fluoroquinolone resistance testing in 30% of the positive *M. genitalium* samples due to limited funding.

In conclusion, we revealed the relatively low prevalence of *M. genitalium* in a large GP population in the Netherlands, but, co-occurrence with *C. trachomatis* and resistance to azithromycin may be observed. Therefore, when treating STIs, these prevalence and resistance data should be taken into account.

## Data Availability

The data that support the findings of this study are available on request from the corresponding author.
